# Spatiotemporal dynamics of malaria in Banmauk Township, Sagaing region of Northern Myanmar: characteristics, trends, and risk factors

**DOI:** 10.1186/s12879-022-07634-6

**Published:** 2022-07-28

**Authors:** Pyae Linn Aung, Myat Thu Soe, Thit Lwin Oo, Kyaw Thu Aung, Kyaw Kyaw Lin, Aung Thi, Lynette Menezes, Daniel M. Parker, Liwang Cui, Myat Phone Kyaw

**Affiliations:** 1 Myanmar Health Network Organization, Yangon, Myanmar; 2 Township Health Department, Banmauk Township, Sagaing, Myanmar; 3 Department of Public Health, Ministry of Health, NayPyiTaw, Myanmar; 4 Division of Infectious Diseases and International Medicine, Department of Internal Medicine, Morsani College of Medicine, University of South Florida, 3720 Spectrum Boulevard, Suite 304, Tampa, FL 33612 USA; 5 Department of Population Health and Disease Prevention, University of California, Irvine, CA USA

**Keywords:** Malaria, Epidemiology, Annual Parasite incidence, Spatial distribution, Severe malaria, Risk factor, Northern Myanmar

## Abstract

**Background:**

While national malaria incidence has been declining in Myanmar, some subregions within the nation continue to have high burdens of malaria morbidity and mortality. This study assessed the malaria situation in one of these regions, Banmauk Township, located near the Myanmar-India border. Our goal was to provide a detailed description of the malaria epidemiology in this township and to provide some evidence-based recommendations to formulate a strategy for reaching the national malaria elimination plan. Banmauk consistently has one of the highest malaria burdens in Myanmar.

**Methods:**

With the implementation of strengthened malaria control and surveillance activities after the endorsement of a national malaria elimination plan in 2015, detailed incidence data were obtained for 2016–2018 for Banmauk Township. The data include patient demographics, parasite species, disease severity, and disease outcome. Data were analyzed to identify characteristics, trends, distribution, and risk factors.

**Results:**

During 2016–2018, 2,402 malaria cases were reported, with *Plasmodium falciparum* accounting for 83.4% of infections. Both *P. falciparum* and *P. vivax* were transmitted more frequently during the rainy season (May–October). Despite intensified control, the annual parasite incidence rate (API) in 2017 (11.0) almost doubled that in 2016 (6.5). In total, 2.5% (59/2042) of the cases, of which 54 *P. falciparum* and 5 *P. vivax*, were complicated cases, resulting in 5 deaths. Malaria morbidity was high in children < 15 years and accounted for 33.4% of all cases and about 47% of the complicated cases. Older age groups and males living with poor transportation conditions were more likely to test positive especially in rainy and cold seasons. Despite the clear seasonality of malaria, severe cases were found among young children even more common in the dry season, when malaria incidence was low.

**Conclusions:**

Despite the declining trend, the malaria burden remained high in Banmauk Township. Our study also documented severe cases and deaths from both falciparum and vivax malaria. *P. falciparum* remained the predominant parasite species, demanding increased efforts to achieve the goal of elimination of *P. falciparum* by 2025. As *P. falciparum* cases decreased, the proportion of cases attributable to *P. vivax* increased. In order to eliminate malaria, it will likely be important to increasingly target this species as well.

## Background

Compared with 2010, Myanmar has made remarkable progress in controlling malaria, with the 2018 data showing 31.8% and 97.6% reductions in morbidity and mortality, respectively [1]. However, with 70,432 malaria cases and 19 deaths in 2018, Myanmar continues to have the highest malaria-burden of any country in the Greater Mekong Sub-region (GMS) [1]. Within Myanmar, malaria case distribution is highly uneven with cases mostly occurring in hard-to-reach rural areas with limited health facilities, shortages of resources for control, and in conflict zones [2]. In 2018, most malaria cases were found in townships of five states and regions: Chin, Kayin, Rakhine, Kachin, and Sagaing [3]. With the endorsement of a national strategic plan for malaria elimination in 2015 [3, 4], national malaria control activities have geared up especially expanding the diagnosis and treatment capabilities, along with the disease surveillance system, in collaboration with multiple national and international partners [4, 5]. In order to achieve malaria elimination in the targeted timeframe, all malarious regions in the country are coordinating their efforts to reduce the disease burden in collaboration with their adjacent areas.

There are multiple challenges to the malaria elimination campaign in Myanmar. First, drug resistance, especially the emergence of artemisinin resistance in *Plasmodium falciparum* reported in eastern and southern Myanmar [6, 7], may compromise the efficacy of artemisinin-based combination therapies (ACTs) for *P. falciparum* cases. Even though the proportion of *P. falciparum* has substantially reduced in the eastern part of Myanmar, the 2018 country-wide data still described 55.4% of the malaria cases attributing to *P. falciparum* [1]. To mitigate the drug resistance problem, the national malaria treatment guidelines (NMTG) have been updated and more stringent patient guidelines are being pursued [8]. Refresher trainings on malaria diagnosis and treatment have been conducted to promote the capabilities of malaria healthcare providers, government healthcare personnel, and community-level malaria volunteers to ensure early diagnosis and effective treatment [9]. In addition, the clinical efficacy of antimalarial drugs has been closely monitored in sentinel sites across Myanmar. Second, malaria prevention measures in vulnerable populations are still inadequate in remote areas. For residents in areas with ongoing malaria transmission, a key preventive measure is the use of long-lasting insecticide-treated nets (LLINs). However, LLIN utilization remains insufficient; LLIN ownership is low in many rural communities [10, 11]. Also, appropriate vector control measures remain limited and need to be strengthened [12]. Third, an improved malaria surveillance system is needed to accurately and timely identify transmission hotspots and deliver targeted control efforts [13, 14]. The current surveillance system fails to reach underserved populations. Fourth, delays in receiving early diagnosis and effective treatment may also lead to more severe disease outcomes, especially among *P. falciparum* patients. To reduce unnecessary deaths from malaria, it is important that early diagnosis and treatment be achieved from both provider and a consumer viewpoints. Surveillance activities at the township level remains inadequate [15]. Other challenges include insufficient resources, questionable performance of village health volunteers (VHVs), lack of a strong evidence-base for implementation of interventions, and low uptake of interventions among community members all impede the progress towards malaria elimination [3, 16, 17].

Although malaria control activities have been implemented, evaluation of the effectiveness of these control activities remains scarce. The current study aims to describe the current malaria situation, and to provide evidence-based recommendations to formulate future implementation strategies, in Banmauk and other high-burden settings like it in Myanmar. Rapid reduction of malaria in these settings could help move the country towards achieving nationwide malaria elimination in the planned timeframe.

## Methods

### Study site

**Fig. 1:**
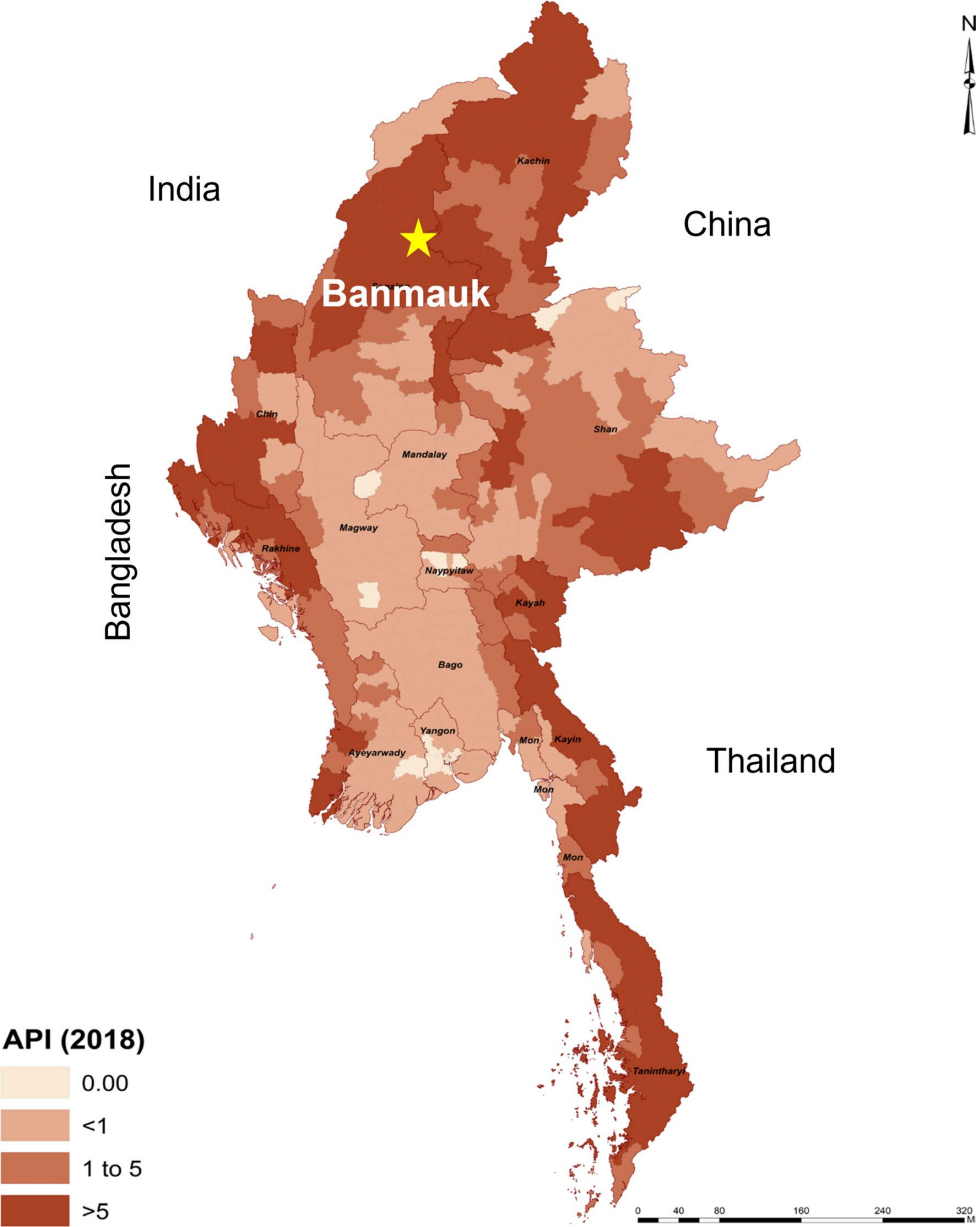
Map of Myanmar showing the location of Banmauk Township, overlaid on the 2018 nation-wide API

To track the progress towards malaria elimination in Myanmar, Banmauk Township was purposively selected for an epidemiological investigation to provide recommendations for malaria elimination at the township level. Banmauk is located in the Sagaing Region in the north, close to the Myanmar-India border (Fig. 1), with a population of 112,668 according to the 2014 census. It is among the top 10 high-burden townships based on the 2018 malaria distribution in Myanmar. Therefore, the township is still in the transmission reduction phase, and only malaria control-specific activities are delivered by different organizations, including the government vector-borne disease control team (VBDC). The activities include but are not limited to malaria surveillance and treatment through trained health staff and malaria volunteers, active case detection by mobile clinics, village-based health education, and distribution of insecticidal bed-nets. Malaria elimination-specific activities such as case investigation, foci investigation, and response are not yet implemented.

A total of 219 villages received oversight healthcare services provided by one township hospital, 2 station hospitals, 8 rural health centers and 37 rural health facilities in Banmauk. Around 150 basic healthcare providers were assigned by the government through the Ministry of Health. Malaria control is coordinated and supervised by the VBDC team. The team has trained and supported 30 VHVs in high-risk villages, which do not have rural health facilities serving these villages. In addition to VBDC, other non-governmental malaria partners (Myanmar Medical Association, Population Services International, and the Malaria Consortium) also provided services in Banmauk Township from 2016 to 2018. Their participation was coordinated by the VBDC team to provide uniform coverage in the target villages.

The township has a subtropical climate, and the months can be divided into dry (November–April) and wet (May–October) seasons. Temperatures are warm throughout the year with a mild winter (December–February). In 2019, mean ambient temperature ranged from 9 to 35 °C, and the cumulative precipitation was 27.02 inches. It is an agricultural region and most of the population are farmers. Many informal gold mines exist in the region.

### Malaria diagnosis and treatment

Malaria control activities are delivered in the community in line with the national strategy. Malaria diagnosis relies mainly on a WHO-prequalified rapid diagnostic test (RDT) for *P. falciparum* and *P. vivax* (SD BIOLINE Malaria Ag *P.f*/ *P.v* test). The overall sensitivity and specificity of these RDTs for *P. falciparum* is 99.7% and 99.5% and for *P. vivax*, it is 95.5% and 99.5%, respectively. Diagnosis by microscopy was only available at the township hospital. While all five species of malaria that naturally infect humans have been identified in Myanmar, the RDTs being used for diagnosis are only capable of identifying falciparum or vivax malaria infections.

The current treatment for uncomplicated falciparum malaria is artemether-lumefantrine (AL) plus single-dose primaquine (PQ) (0.75 mg/kg). For vivax malaria, it is chloroquine-primaquine. *P. falciparum* and *P. vivax* mixed infections are treated by AL and 14 days of primaquine (0.25 mg/kg/day). Routine G6PD testing among patients with malaria cannot be implemented yet. Still, they were asked to check urine color during PQ treatment and seek immediate follow-up care if they found red-colored urine. Other hypnozoidal drugs like Tafenoquine are still under trial to use in Myanmar. However, in some urban townships of central Myanmar with few malaria cases, directly observed treatment for PQ either by a health care provider or a family member has been introduced.

At the village level, health care providers (e.g., health assistant, midwife, or public health staff-II) are based at each health center and are responsible for malaria testing, treatment, and delivering prevention services. They were well-trained for managing malaria during their school times and are regularly refreshed for the updated changes and data entry guidelines by the township VBDC team. Patients presenting with signs and symptoms of complicated or severe malaria, as assessed by field healthcare providers, are referred to the nearest hospitals. Here we define severe malaria as individual presence of *Plasmodium* parasitemia either *P. falciparum* or other species and showed one or more manifestations of complicated or severe malaria [9, 18]. As a pre-referral treatment and life-saving measure, an initial dose of injectable artesunate is administered without delay [18]. However, as resistance to artesunate has been reported in Myanmar, intravenous Quinine is used as a second-line treatment for both severe malaria and pregnant women during their early trimesters. Malaria prevention includes mass distribution of LLINs with annual supplemental distribution to high-risk individuals including young children and pregnant women. Indoor residual spraying of insecticide is not conducted.

### Data collection and entry

Each health center used standard forms of patients’ registries issued by the VBDC. Data entry guidelines were disseminated and updated as necessary through monthly meetings at township hospitals. Initial data collection was done using a nationally standardized paper-based case report form (CRF), which includes the patient’s demographic information (name, age, sex, and address), history of present illness, blood test results, and treatment.

At the time of diagnosis and treatment, the respective health staff at each rural health facility filled in the carbonless CRF for each malaria-suspected individual. The CRFs were transferred to the township VBDC office monthly to enter a cloud database using the DHIS-II desktop application. If potential mistakes were detected in the CRFs by the data verifier at the township level, the person filing the initial form was contacted for clarification and correction. An initial was required for each correction. The data entry person assigned by the WHO at the state and regional health units regularly checked and ensured data correctness, completeness and free of any missing values entered at the township level. The updated versions of monthly data files were uploaded to cloud storage after data confirmation.

In this study, only the finalized data from cloud storage were downloaded and used by the researchers in late 2019 after obtaining appropriate approval from the township health department. Monthly malaria incidence data were combined into a single Excel file, a random countercheck on data correctness and accuracy was performed by the researchers, and cases with incomplete variables were excluded during the analysis. Only two cases with no malaria diagnostic results were observed and excluded.

### Exploratory temporal and spatial analyses

The overall malaria trend was assessed using two metrics – the test positivity ratio, which measures the proportion of confirmed malaria cases of total suspected cases each year calculated by number of tests positive divided by number of tests done, and the annual parasite incidence (API), which measures the number of confirmed malaria incidence per 1,000 population in a given year.

We then mapped the case incidence (or annual parasite incidence (or API)) at the township level for each year of the study period (2016, 2017, and 2018). Maps were generated separately for all malaria, for *P. vivax*, *P. falciparum*. We also plotted severe cases (using points) on top of the map of malaria API.

We then used scan statistics to detect the most likely clusters of *P. falciparum*, *P. vivax*, and severe malaria cases over time. For the analysis, we used a discrete-time, space-time Poisson model [19]. Geographic centroids were calculated for each of the village tracts. The data were aggregated by month (total of 36 consecutive months, from January 2016 through December 2018). The scan statistic uses a moving spherical window that centers on each geographic centroid and compares the observed number of *P. falciparum*, *P. vivax*, or severe malaria cases to the expected number based on the population size of that window and the distribution of cases and population across the entire study area. The window increases in size until half of the township population is contained and then moves to the next point. Likelihood ratios were calculated for each window location, size, and time point. P-values were calculated using Monte Carlo simulations for the largest ranking clusters. The results of this analysis provide the general locations of likely space-time clusters of malaria cases, given the distribution of cases and population across the study location and study time period.

### Statistical analysis of individual predictors of falciparum, vivax, and severe malaria

Logistic regressions were then used to analyze risk factors for *P. falciparum* or *P. vivax* infection, as well as for severe malaria outcomes. For the risk factors analysis for *P. falciparum* and *P. vivax* malaria, patients with no malaria infection were used as the referent group. For the analysis of severe malaria, uncomplicated cases were considered as referent group. We hypothesized that age, sex, season, and transportation capabilities would be predictive of infections and of severe outcomes. We therefore included these variables in all multivariable models.

### Software

Graphs were produced using Microsoft Excel 2018 (Excel for Mac, version 16.16.27, Seattle, WA, USA). Scan statistics were calculated using SaTScan software (https://www.satscan.org/). Maps were created using QGIS version 3.12. The logistic regressions were done using the Statistical Package for the Social Sciences (IBM SPSS Statistics for Macintosh, version 23, IBM Corp., Armonk, NY, USA).

### Ethics declaration

This study was reviewed and approved by the Institutional Review Boards of the Department of Medical Research, Myanmar (Ethics/ DMR/ 2017/ 077AE/ 2018E/ 2019E/ 2020), and the University of South Florida. All methods were carried out in accordance with relevant guidelines and regulations.

## Results

### Overall trend of malaria in Banmauk

**Fig. 2:**
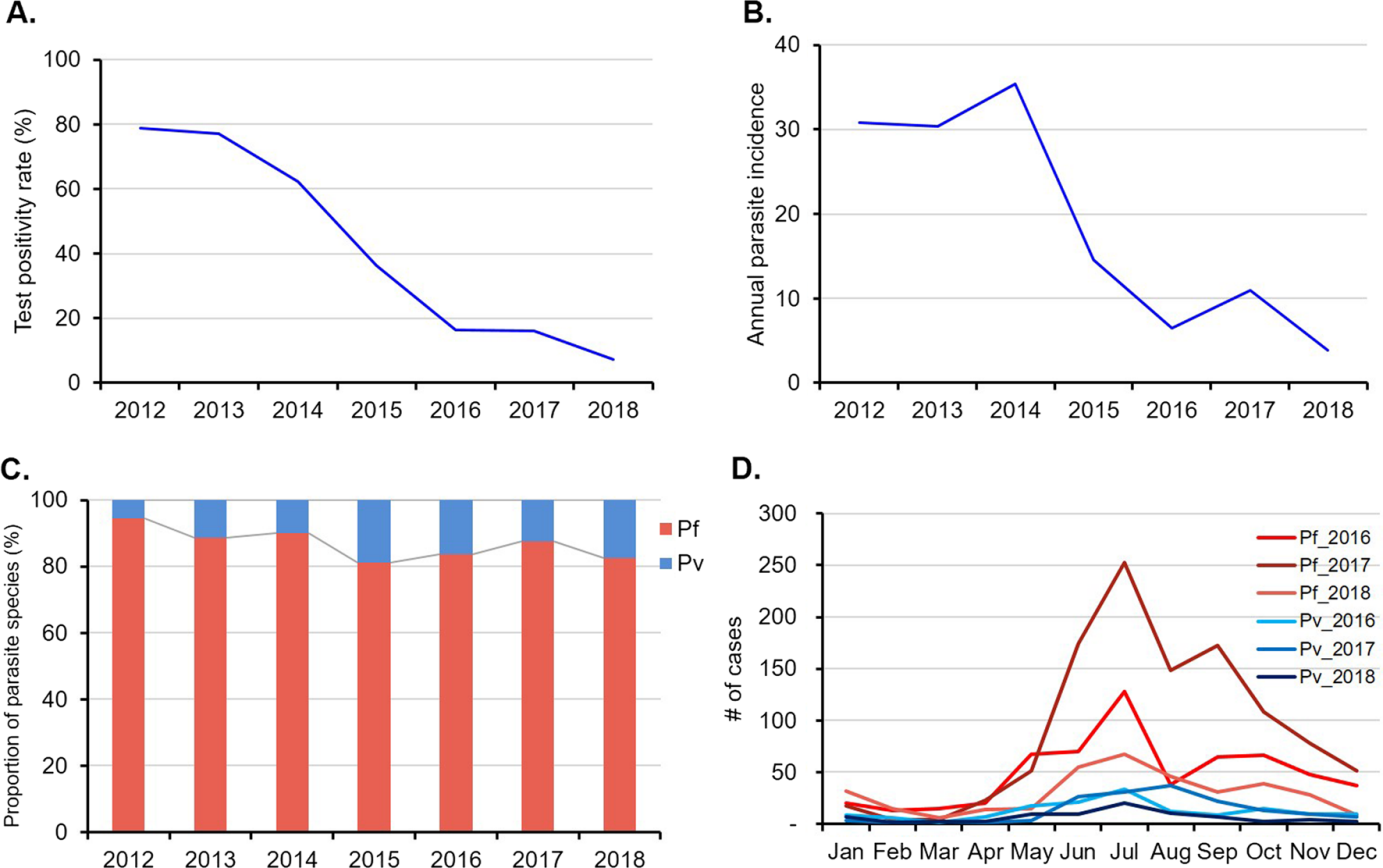
Overall trend of malaria in Banmauk. **A** Test positivity rate (2012–2018). **B** Annual parasite incidence rate (2012–2018). **C** *Plasmodium* species composition (2012–2018). **D** Malaria seasonality (2016–2018)

Banmauk is among the townships with the highest malaria burden in Myanmar. In 2017, Banmauk had an API of 11.0 as compared to a country-wise API of 1.6. Among the 219 villages of Banmauk, 87.0% were grouped in the high-risk category (API > 5). Compared to a test positivity of almost 80.0% in 2012, the test positivity continuously declined and reached 7.3% in 2018 (Fig. 2A). Meanwhile, there was an almost 8-fold reduction in the annual API, from 30 to 2012 to 3.8 in 2018, although fluctuations were detected in 2014 and 2017 (Fig. 2B).

Since Myanmar’s stated goal of working toward malaria elimination in 2015, strengthened surveillance has been implemented and the data in 2016–2018 were analyzed in more detail. In these three years, a total of 18,061 suspected malaria cases were tested, of which 13.3% were confirmed malaria cases (Table 1). The positivity ratios also showed a more than 2-fold decrease from 16.2% to 2017 to 7.3% in 2018. The major fluctuation in 2016–2018 in confirmed malaria cases also corresponded to a similar fluctuation in API (Table 1; Fig. 2B). Among the 2,402 *Plasmodium*-positive patients, 97.5% presented with uncomplicated malaria and were treated as outpatients. A total of 59 patients (2.5%) presented with severe symptoms and five of them died from malaria in 2016 and 2017 (Table 1).

**Table 1:** Malaria epidemiology and trend in Banmauk Township (2016–2018)

	2016	2017	2018	Total
Case description				
Total suspected cases tested	4492	7653	5916	18,061
Confirmed malaria cases	733	1,238	431	2402
Positivity rate (%)	16.3	16.2	7.3	13.3
API*	6.5	11.0	3.8	7.1
Disease conditions [n (%)]				
Non–severe	717 (97.8)	1,197 (96.7)	429 (99.5)	2,343 (97.5)
Severe	16 (2.2)	41 (3.3)	2 (0.5)	59 (2.5)
Deaths	3 (0.41)	2 (0.16)	0	5 (0.21)
Malaria parasite [n (%)]				
* P. falciparum*	599 (81.7)	1,052 (85.0)	352 (81.7)	2,003 (83.4)
* P. vivax*	120 (16.4)	154 (12.4)	75 (17.4)	349 (14.5)
Mixed infections	14 (1.9)	32 (2.6)	4 (0.9)	50 (2.1)

*API (incidence per 1000 population) was based on 2014 nation-wide population census

### ***Plasmodium*species composition**

In this remote area, malaria diagnosis was mostly based on an RDT, and thus only *P. falciparum* and *P. vivax* infections were differentiated. In the past decade, *P. falciparum* remained the predominant species, constituting more than 80.0% of total *Plasmodium* infections (Fig. 2C). During 2016–2018, *P. falciparum* accounted for 83.4% of total infections while only 2.1% presented mixed infections (Table 1).

### Malaria seasonality

As in other subtropical and tropical regions of SE Asia, malaria transmission in Banmauk is perennial but showed clear seasonality (Fig. 2D). Analysis of the three-year data in 2016–2018 revealed that malaria incidence was low in the cold and dry months (January–April) and began to rise at the onset of the rainy season in May and peaked in July. *P. falciparum* infections showed a second, smaller peak in September or October. *P. vivax* showed a similar seasonal trend, but the second peak was not always apparent.

### Spatial heterogeneity and temporal changes

**Fig. 3:**
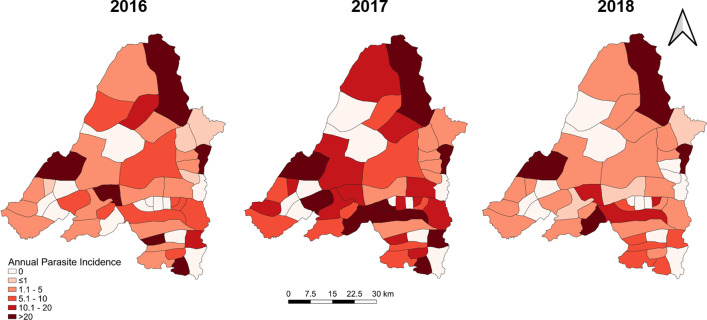
Spatial distribution of overall malaria prevalence in Banmauk in 2016–2018

Banmauk Township is divided into 48 subcenters based on the presence of a health facility, and the subcenters have been classified into high-, moderate-, and low-risk categories corresponding to an API of > 10, 1–10 and < 1, respectively. Choropleth API maps of the subcenters displayed significant heterogeneity, with high-risk subcenters intermingled with low- or moderate-risk ones (Fig. 3). Over the three years, the low risk subcenters remained stable and the fluctuation mostly reflected changes between the high and moderate categories. In 2016, 17 and 14 subcenters were in the high- and moderate-risk categories, respectively. However, in 2017, the number of high-risk subcenters increased to 26, primarily due to the transition of subcenters from the moderate- to the high-risk category. With the improvement of the situation in 2018, the number of high-risk centers decreased to 11 (Fig. 3). Most of the areas near the township center, where the township hospital is located, were in the high-risk category in 2017, while the peripheral villages showed progressively decreasing API. Further, most high-risk subcenters include populated village tracts and forest areas. Some are the locations of goldmines, where the miners lived in makeshift shelters with less protection against malaria (Fig. 3).

**Fig. 4:**
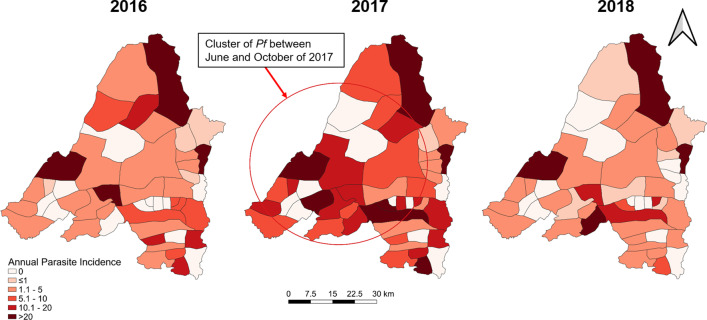
Distribution of severe malaria cases in 2016 and 2017 overlaid on the API of individual subcenters. The cluster of severe malaria is highlighted by a circle

Interestingly, there was apparent clustering of the severe cases in 2017, with most of them (31.7%) originating from villages within 5 km of the Banmauk town center (Fig. 4). The distribution of severe cases was disproportionate to the overall malaria distribution during 2016 and 2017, as many of the severe cases were from low risk subcenters. In addition, another cluster of severe cases (28.3%) was from an area about 20 km southeast of the town (Fig. 4). These two areas were conducive for malaria transmission with forest backgrounds and clustered gold mines.

**Fig. 5:**
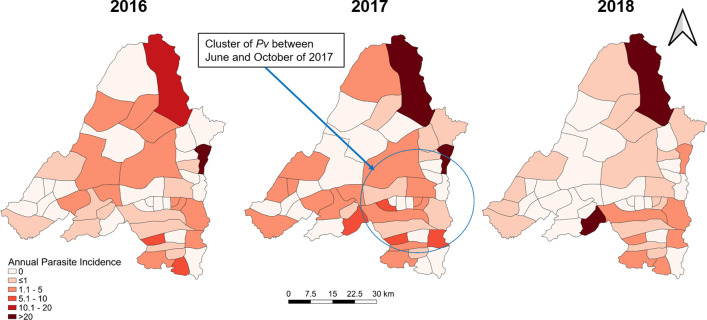
Spatial distribution of *P. falciparum* in Banmauk 2016–2018. The cluster of *P. falciparum* is highlighted by a circle

**Fig. 6:**
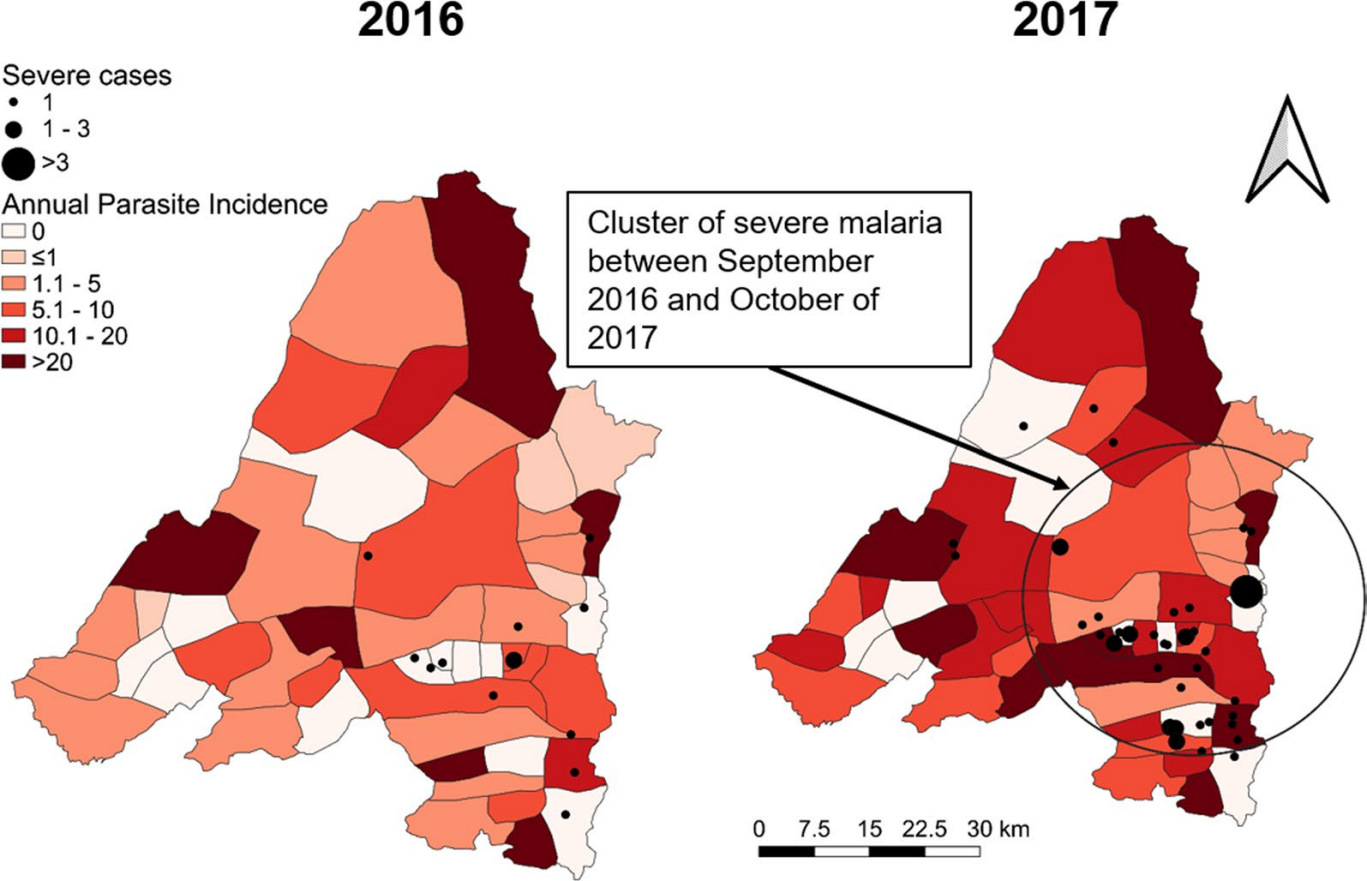
Spatial distribution of *P. vivax* in Banmauk 2016–2018. The cluster of *P. vivax* is highlighted by a circle

Scan statistics identified separate, partially overlapping clusters of *P. falciparum*, *P. vivax*, and severe malaria cases at different points in time (Figs. 5, 6 and 4). *P. falciparum* cases clustered in the Western portion of the township from June through October 2017 (relative risk (RR) of 5.20). A cluster of *P. vivax* cases was also found during the same time period but in the Southeastern portion of the township (RR of 5.09). Finally, a cluster of severe malaria cases was identified in the eastern portion of the township (overlapping the *P. vivax* cluster) from September 2016 through October 2017 (RR of 17.08).

### Risk factors associated with malaria test positivity

Factors potentially associated with malaria test positivity of each species were estimated (Table 2). Males had significantly higher odds than females in being diagnosed with either *P. falciparum* (AOR: 2.0, 95% CI: 1.80–2.23) or *P. vivax* infections (AOR: 1.83, 95%CI: 1.44–2.32). Older age groups had significantly increased odds of *P. falciparum* infections. Seasonal variations also influenced the test positivity of *P. falciparum* and *P. vivax* infections. Rainy season and cold season showed higher odds than that of dry season for both *P. falciparum* and *P. vivax*. People living with poor transportation had significantly higher odds than those with good transportation condition in being diagnosed with either *P. falciparum* (AOR: 7.13, 95% CI: 6.31–8.06) or *P. vivax* infections (AOR: 6.05, 95%CI: 4.64–7.87) (Table 2).

**Table 2:** Underlying risk factors of malaria infection (2016–2018) (n = 18,061)

Descriptions	*P. falciparum* cases (n = 2053)				*P. vivax* cases (n = 349)			
	*P. falciparum* cases n (%)	Non–*P. falciparum*/ no infection n (%)	COR (95%CI)	AOR (95% CI)	*P. vivax* cases n (%)	Non–*P. vivax*/ no infection n (%)	COR (95%CI)	AOR (95% CI)
Sex								
Female	710 (34.6)	7392 (46.2)	1*	1	119 (34.1)	7983 (45.1)	1*	1
Male	1343 (65.4)	8616 (53.8)	1.62 (1.47–1.79)	2.00 (1.80–2.23)	230 (65.9)	9729 (54.9)	1.59 (1.27–1.98)	1.83 (1.44–2.32)
Age (years)								
< 5	202 (9.8)	3424 (21.4)	1*	1	53 (15.2)	3573 (20.2)	1	1
5–14	467 (22.7)	4062 (25.4)	1.95 (1.64–2.31)	1.79 (1.51–2.12)	82 (23.5)	4447 (25.1)	1.24 (0.88–1.76)	1.16 (0.82–1.64)
15–24	602 (29.4)	3274 (20.5)	3.12 (2.64–3.68)	2.46 (2.07–2.92)	87 (24.9)	3789 (21.4)	1.55 (1.10–2.18)	1.15 (0.81–1.64)
≥ 25	782 (38.1)	5248 (32.8)	2.53 (2.15–2.97)	1.86 (1.56–2.22)	127 (36.4)	5903 (33.3)	1.45 (1.05–2.00)	0.94 (0.67–1.31)
Seasonal variation								
Dry season (Jan–Apr)	310 (15.1)	2954 (18.5)	1*	1	57 (16.3)	3207 (18.1)	1*	1
Rainy season (May–Sep)	1,092 (53.2)	8652 (54.0)	1.20 (1.05–1.37)	1.63 (1.41–1.87)	169 (48.4)	9575 (54.1)	0.99 (0.73–1.34)	1.21 (0.89–1.66)
Cold season (Oct–Dec)	651 (31.7)	4402 (27.5)	1.41 (1.22–1.63)	3.69 (3.11–4.39)	123 (35.2)	4930 (27.8)	1.40 (1.02–1.93)	3.16 (2.21–4.52)
Transportation conditions^a^								
Good	591 (28.8)	9776 (61.1)	1*	1	101 (28.9)	10,266 (58.0)	1*	1
Poor	1462 (71.2)	6232 (38.9)	3.88 (3.51–4.29)	7.13 (6.31–8.06)	248 (71.1)	7446 (42.0)	3.39 (2.68–4.27)	6.05 (4.64–7.87)

^a^The transportation was grouped according to the roads condition regardless of the distance between patient’s place and township hospital: good: can go easily by any vehicles in all seasons, poor: the route consumed prolonged time or have to take both vehicles and foot even near or far; *p-value < 0.05 as determined by chi-square test; *COR* Crude odds ratio, *AOR* Adjusted odds ratio, *CI* Confidence Interval

Underlying risk factors related with overall malaria infection were also explored (Table 3). Male possessed higher odds than female (AOR: 1.58, 95% CI: 1.43–1.73). Malaria infection is more vulnerable among older age > 5 years. Overall malaria infection is also common during rainy season (AOR: 1.17, 95% CI: 1.03–1.33) and cold season (AOR: 1.2, 95% CI: 1.04–1.38) than that of dry season. People living with poor transportation conditions were more likely to test positive than people with good transportation access (AOR: 4.28, 95%CI: 4.25–5.31) (Table 3).

**Table 3:** Risk factors for malaria infection and severe malaria (2016–2018)

Descriptions	Malaria cases (n = 2402)	No infection (n = 15,659)	COR (95%CI)	AOR (95%CI)	Severe cases (n = 59)	Non–severe cases (n = 2343)	COR (95%CI)	AOR (95%CI)
	**n (%)**	**n (%)**			**n (%)**	**n (%)**		
Age (years)								
<5	255 (10.6)	3371 (21.5)	1*	1	18 (30.6)	237 (10.1)	4.53 (2.25–9.12)	4.31 (2.12–8.77)
5–14	549 (22.9)	3980 (25.4)	1.82 (1.56–2.13)	1.81 (1.55–2.12)	10 (16.9)	539 (23.0)	1.11 (0.49–2.48)	0.63 (0.17–2.37)
15–24	689 (28.7)	3187 (20.4)	2.86 (2.46–3.33)	2.82 (2.42–3.28)	16 (27.1)	673 (28.7)	1.42 (0.70–2.89)	1.03 (0.40–2.66)
≥25	909 (37.8)	5121 (32.7)	2.35 (2.03–2.71)	2.40 (2.07–2.79)	15 (25.4)	894 (38.2)	1*	1
Sex								
Female	829 (34.5)	7273 (46.4)	1*	1	27 (45.8)	802 (34.2)	1	1
Male	1573 (65.5)	8386 (53.6)	1.65 (1.50–1.80)	1.58 (1.43–1.73)	32 (54.2)	1,541 (65.8)	0.62 (0.37–1.04)	0.67 (0.38–1.14)
Seasonal variation								
Dry season (Jan–Apr)	367 (15.3)	2897 (18.5)	1*	1	25 (42.4)	733 (31.3)	1	1
Rainy season (May–Sep)	1261 (52.5)	8483 (54.2)	1.17 (1.04–1.33)	1.17 (1.03–1.33)	21 (35.6)	990 (42.3)	0.62 (0.35–1.12)	0.69 (0.32–1.51)
Cold season (Oct–Dec)	774 (32.2)	4279 (27.3)	1.43 (1.25–1.63)	1.20 (1.04–1.38)	13 (22.0)	620 (26.4)	0.62 (0.31–1.21)	0.54 (0.28–1.02)
Transportation conditions^a^								
Good	692 (28.8)	9675 (61.8)	1*	1	20 (33.9)	922 (39.4)	1	1
Poor	1710 (71.2)	5984 (38.2)	4.0 (3.64–4.39)	4.28 (4.25–5.31)	39 (66.1)	1,421 (60.6)	1.27 (0.73–2.18)	0.74 (0.18–1.62)
Parasite species								
* P. falciparum*	–	–	–	–	54 (91.5)	1,999 (85.3)	1.86 (0.74–4.68)	1.86 (0.73–4.75)
* P. vivax*	–	–	–	–	5 (8.5)	344 (14.7)	1	1

For analysis between severe and non-severe cases, uncomplicated cases or non-severe cases were considered as referent group; ^a^The transportation was grouped according to the roads condition regardless of the distance between patient’s place and township hospital: good: can go easily by any vehicles in all seasons, poor: the route consumed prolonged time or have to take both vehicles and foot even near or far; *p-value < 0.05 as determined by chi-square test; *COR* Crude odds ratio, *AOR* Adjusted odds ratio, *CI* Confidence Interval

### Severe malaria cases, outcomes, and risk factors

In 2016–2018, 2.5% (59/2,402) of the malaria cases exhibited signs and symptoms of severe malaria and were treated as inpatients in the township hospital (Table 4). Based on the NMTG, all patients were treated with intravenous artesunate injection. The majority (91.5%, 54/59) of these severe cases were due to *P. falciparum*. For the 2,003 *P. falciparum* cases, 2.7% were severe malaria cases, with most of the signs or symptoms presented as convulsions (29.6%), coma (24.1%), and respiratory distress (20.4%). Though *P. vivax* has been considered a benign malaria, 1.4% (5/349) of *P. vivax* cases in these data had accompanying severe symptoms, including convulsion, jaundice, and coma. Whereas 54 patients with complicated malaria eventually recovered, five cases were fatal – four were due to *P. falciparum* and one due to *P. vivax*. One of the deaths occurred in a child with *P. falciparum*. The deaths were due to unrecovered shock (2 cases), renal failure (2 cases), and severe respiratory distress (1 case).

**Table 4:** Clinical presentations of severe malaria cases

Signs and symptoms	*Plasmodium falciparum* (n = 54)		*Plasmodium vivax* (n = 5)	
	**n**	**%**	**n**	**%**
Convulsions	16	29.6	2	40.0
Coma	13	24.1	1	20.0
Respiratory distress	11	20.4	–	–
Renal failure	8	14.8	–	–
Jaundice	–	–	2	40.0
Prostration	4	7.4	–	–
Shock	2	3.7	–	–

Severe malaria cases were significantly more frequent (30.6%) in children under 5 years of age (Table 3). Compared to older age as the reference group, however, children < 5 years bore significantly higher risks of severe malaria (AOR: 4.31, 95% CI: 2.12–8.77). While severe malaria occurred more commonly in the wet season, a large proportion (42.4%) of severe cases occurred in the dry season (January–April). There were higher odds of severe cases among patients with *P. falciparum* infection (AOR: 1.86, 95%CI: 0.73–4.75) (Table 3).

## Discussion

This study aimed to describe malaria epidemiology in a remote township in Northern Myanmar with a major burden of malaria (Banmauk Township). While malaria incidence showed an overall trend of decline over the last decade in Banmauk, the 2017 malaria incidence almost doubled that of 2016. One reason for this increase might be due to increased test capacity in 2017, when there was a 31.6% increase of febrile cases being tested for malaria infections. Another reason might be due to increased malaria incidence in underserved populations including migrants who reside in the forest or mining area. Compared to 2016, several private mining companies entered Banmauk in 2017, associated with increased mobile and migrant populations.

*P. falciparum* tends to be more responsive to intensified control than *P. vivax* (because of the complications associated with radical cure of vivax malaria), and vivax malaria has become the predominant species in many parts of the GMS [20, 21]. In Banmauk *P. falciparum* has remained the predominant parasite species responsible for over 80% of all malaria cases. The persistence of falciparum malaria in this region may be due to several reasons. The proximity of many villages to forested areas may mean that ecological conditions are conducive to higher malaria transmission [22, 23]. Changes in vector species composition as the result of environmental changes has been considered responsible for a shift in malaria parasite species prevalence observed in eastern Thailand [24], highlighting the importance of the environment-vector-parasite inter-relationship. Another reason could be emerging artemisinin resistance. Though ACTs were found to be highly efficacious in other regions of upper Myanmar [25, 26], there were indications of increased prevalence of pfk13 mutations associated with artemisinin resistance in northern Myanmar [25, 27]. Banmauk has been categorized as a malaria control area, and therefore provided low budget allocation. A follow-up assessment of ACT efficacy has therefore not been performed. Given that such information is deemed highly important for delivering effective management of malaria cases, it may be logistically feasible to use day-3 parasitemia and pfk13 mutations as the proxy for artemisinin resistance in this area [28]. Furthermore, the use of registered drugs should be appropriately regulated, and treatment compliance should also be monitored [3].

Like in other GMS regions, malaria in Banmauk has clear seasonality, with 71.0% of malaria cases occurring from June to September. It coincides with the increased abundance of malaria vectors during the wet season from entomologic studies [29, 30]. Currently, the biology and behaviors of malaria vectors in this part of Myanmar remain poorly studied and understood. Vector-based preventive measures are based on LLINs alone. Although LLINs have been widely distributed in Banmauk, their coverage and utilization were not followed. More vulnerable populations, including migrants associated with the gold mines, are overlooked since LLIN distribution is mostly based on the village registry. LLINs are also most protective indoors and during nighttime hours and diseases that are spread by mosquito vectors that feed outdoors or at dusk or dawn may not be impacted by LLIN use. Additional vector-based control measures, such as residual spraying of insecticide, should be considered. Moreover, during the rainy season, even a short distance from the health center may cause significant delays in treatment-seeking [31]. The establishment of village-level malaria posts through VHVs may play a crucial role in timely diagnosis and treatment. In the case of Banmauk, most malaria cases (91.0%) were treated by field healthcare personnel, either village health volunteers or basic health staff, under the supervision of respective health assistants. Thus, this group of base-level healthcare providers needs to be vigorously trained and to receive refresher training regularly so that they can deliver high-quality management of malaria cases at the village level. Supportive supervision should be conducted to ensure their accomplishments. Full availability of diagnostic tools and antimalarial drugs should be steadily secured for these village-level malaria posts.

As demonstrated in other studies, age and sex are major determinants for contracting malaria [32, 33]. The age distribution of malaria is strongly influenced by the intensity of malaria transmission. In hyperendemic areas, children made up the major proportion of clinical cases and severe cases [34], whereas, in low transmission settings, adults often carry higher occupation-related risks. In the low-endemicity settings of Banmauk, the older age groups ≥ 5 years all were associated with significantly increased risks of malaria infections. The increased risks of malaria infections in adults may be due to their forest- and agriculture-related occupations [32, 33]. Though increased risks of the school-aged children are not completely understood, children in this age group may visit playgrounds and return home after the mosquito biting time [35]. These differences in age distribution between the two regions that are only ~ 200 km apart and between the two different parasite species suggest that the different disease ecologies and vector species may be the underlying determinant factors of the different malaria transmission patterns. Nevertheless, this study revealed that children younger than 15 constituted about 30% of all falciparum cases, and close to 40% of all vivax cases. Moreover, they also accounted for almost 50% of the severe malaria cases. Such high morbidity in children for a low-endemicity area demands more effective preventive care for this vulnerable group. In addition, the proportion of malaria patients was higher among males. Similarly, in northeastern Myanmar, young male adults also had the highest risks of contracting *P. falciparum* malaria [20, 36]. This again might be correlated with forest-related jobs and leisure time outside households at night, which can go beyond the peak mosquito-biting times. In contrast, females are more engaged in household work and have more exposure to daytime health education talks and broadcasts. As a result, women may have higher health literacy and better healthcare seeking behaviors than men [37, 38]. Hence, male-focused interventions or nighttime health talks may help reduce malaria transmission among working-age males.

Another important finding of this study is 2.5% of malaria cases with severe clinical manifestations, with 8% of the severe malaria cases resulting in deaths. Consistent with *P. falciparum* being the predominant parasite (> 80%), *P. falciparum* was also responsible for > 90% (54/59) of the severe malaria cases observed during 2016–2018. Similarly, related studies found severe malaria is more common among *P. falciparum* than *P. vivax* infections [39, 40]. Nevertheless, *P. vivax* infections were also associated with severe conditions or deaths in this and other *vivax*-endemic areas [20]. As discussed above, children < 5 years made up > 30% of the severe cases, deserving more attention. Unexpectedly, while most of the malaria cases occurred in the rainy season, severe malaria cases were even more common in the dry season, when malaria incidence was low (though this finding was not statistically significant). Further, severe cases were spatially clustered and were not proportional to the overall malaria distribution. Interestingly, most severe cases came from the nearest areas from the town where malaria incidence was relatively low. This could be due to poor public awareness of severe malaria in areas where malaria incidence has become rare. In addition, some of the severe malaria clusters occurred in areas with more concentrated gold mines with migrant populations. In many areas, malaria cases were first observed and reported by the field healthcare providers, like the VHVs in the village malaria posts. Therefore, these field workers need to be properly trained to detect early signs of severe malaria and make timely referrals to avert severe and fatal cases. Moreover, RDTs and antimalarial drugs need to be sufficiently supplied. Finally, a proper referral system must be in place to promptly refer severe cases to the nearest hospitals [18].

The current study analyzed malaria incidence data from Banmauk Township in the past decade with focused references to the recent data in 2016–2018. In recognition of the significance of an accurate reporting system for the final phase of malaria elimination, the National Malaria Control Program strived to establish and improve the data reporting system. However, the current surveillance system still has a few weaknesses that need to be improved. The base-level surveillance is delivered by field healthcare workers such as VHVs, who lack some knowledge for malaria diagnosis, treatment and follow-ups and performance incentives [15]. Strengthening their roles in malaria surveillance and prevention would constitute a key foundation activity for the township level VBDC team [41]. In addition, the use of carbonless registers is outdated and does not allow rapid responses to malaria clusters or outbreaks, given that most of the CRFs are delivered to the township health center monthly. The implementation of an electronic case reporting system using cell phones may allow real-time identification of malaria transmission and immediate delivery of malaria control measures proposed for the 1-3-7 strategy. Further, the current surveillance system relied heavily on clinical malaria incidence, and malaria prevalence indicators determined from cross-sectional blood surveys, were not reflected. During the elimination era, WHO recommended achieving an annual blood examination rate of 8 to 10% of populations at risk to confirm the reliability of the indicators [5], which will need to be installed in the top malaria-burden townships.

In summary, the analysis of malaria epidemiology data from a high malaria burden township revealed significant malaria morbidity, the predominance of *P. falciparum* malaria, geographic heterogeneity, and temporal fluctuations in incidence, despite the overall decreasing trend of malaria incidence. The high incidence of *P. falciparum* malaria demands strengthened efforts if the goal to eliminate *P. falciparum* by 2025 is met. The identification of more vulnerable populations encourages the expansion of diagnosis and treatment to reach these high-risk groups. The malaria surveillance system needs to be improved with the active participation of qualified base-level healthcare practitioners and electronic data reporting to ensure real-time responses to malaria cases.

There are several strengths and limitations of this study. The study describes general patterns in, and risk factors for, malaria in a township with one of the heaviest malaria burdens in the region: Banmauk. Our analyses illustrate the usefulness of routine surveillance in evaluating the malaria situation and formulating future strategies, perhaps especially by highlighting significant demographic and geographic risk factors.

Conversely, the data entry only covered the achievements accomplished by government health care personnel and outputs conducted by other nongovernmental organizations were excluded. Likewise only three years of data were available for analysis. Longitudinal data analyses with longer time series would be beneficial. We also lack potentially important socioeconomic data for the population in Banmauk Township. These factors can be important for malaria control and elimination and would be useful for investigation and for consideration in malaria interventions [42]. Finally, it is possible that we missed some clusters of malaria during the time period both because the data were only available in aggregate form (so it is not possible to detect clusters smaller than village tracts) and because of the spherical window that we used in the space-time analysis.

## Conclusions

In this high malaria burden township, the malaria incidence has declined over the past decade but with irregular fluctuations. Targeted health education activities could be implemented among malaria risk groups like adult males living in areas with poor transportation access especially in rainy and cold seasons. To reduce the occurrence of severe malaria cases, it would be beneficial to improve access to early diagnosis and effective treatment, especially for children with *P. falciparum* infection but also for patients with *P. vivax* malaria (while rare, we report severe cases and one death from vivax from this study location). Messaging about the importance of timely diagnosis and treatment should be expanded, and it is important that these messages reach adult males (along with other demographic groups). To reduce unnecessary deaths, the capability of township hospitals and health centers should be increased through increased human resources, frequent trainings, by maintaining the soundness of hospital equipment, and ensuring the availability of anti-malarial drugs and life-saving medicines. Finally, this analysis documents morbidity and mortality from *P. vivax* malaria, which has often been considered a benign disease. Eliminating all malaria from this setting may require approaches that are tailored to this species [43].

## Data Availability

All data analyzed for this study are included within the article.

## Abbreviations

ACTs: Artemisinin-based combination therapies
AL: Artemether and Lumefantrine
AORs: Adjusted odds ratios
API: Annual parasite incidence
CIs: Confidence intervals
CORs: Crude odds ratios
CRF: Case report form
GMS: Greater Mekong Sub-region
LLIN: Long-lasting insecticide-treated net
NMTG: National malaria treatment guidelines
RDT: Rapid diagnostic test
RR: Relative risk
VBDC: Vector-borne disease control
VHV: Village health volunteer
WHO: World Health Organization
