# Correction: Spatiotemporal dynamics of malaria in Banmauk Township, Sagaing region of Northern Myanmar: characteristics, trends, and risk factors

**DOI:** 10.1186/s12879-022-07676-w

**Published:** 2022-08-25

**Authors:** Pyae Linn Aung, Myat Thu Soe, Thit Lwin Oo, Kyaw Thu Aung, Kyaw Kyaw Lin, Aung Thi, Lynette Menezes, Daniel M. Parker, Liwang Cui, Myat Phone Kyaw

**Affiliations:** 1 Myanmar Health Network Organization, Yangon, Myanmar; 2 Township Health Department, Banmauk Township, Sagaing, Myanmar; 3 Department of Public Health, Ministry of Health, NayPyiTaw, Myanmar; 4 Division of Infectious Diseases and International Medicine, Department of Internal Medicine, Morsani College of Medicine, University of South Florida, 3720 Spectrum Boulevard, Suite 304, Tampa, FL 33612 USA; 5 Department of Population Health and Disease Prevention, University of California, Irvine, CA USA

## Correction: BMC Infectious Diseases (2022) 22:653 10.1186/s12879-022-07634-6

Following the publication of the original article [1], we were notified that the figure legends were out of order.

- The label for Fig. 4 should be:
  “Spatial distribution of *P. falciparum* in Banmauk 2016–2018. The cluster of *P. facliparum* is highlighted by a circle.” instead of “Distribution of severe malaria cases in 2016 and 2017 overlaid on the API of individual subcenters. The cluster of severe malaria is highlighted by a circle”.
- The label for Fig. 5 should be:
  “Spatial distribution of *P. vivax* in Banmauk 2016–2018. The cluster of *P. vivax* is highlighted by a circle.” instead of “Spatial distribution of *P. falciparum* in Banmauk 2016–2018. The cluster of *P. falciparum* is highlighted by a circle”.
- The label for Fig. 6 should be:
  “Distribution of severe malaria cases in 2016 and 2017 overlaid on the API of individual subcenters. The cluster of severe malaria is highlighted by a circle.” instead of “Spatial distribution of *P. vivax* in Banmauk 2016–2018. The cluster of *P. vivax* is highlighted by a circle”.

The original article has been corrected.
